# Draft Genome Sequences of Two *Pseudomonas aeruginosa* Bloodstream Infection Isolates Associated with Rapid Patient Death

**DOI:** 10.1128/genomeA.00597-17

**Published:** 2017-08-17

**Authors:** K. L. McCarthy, A. V. Jennison, A. M. Wailan, D. L. Paterson

**Affiliations:** a The University of Queensland, UQ Centre for Clinical Research, Brisbane, Queensland, Australia; b Public Health Microbiology, Forensic and Scientific Services, Queensland Health, Queensland, Australia; c Wellcome Trust Sanger Institute, Hinxton, Cambridge, United Kingdom

## Abstract

The morbidity and mortality associated with *Pseudomonas aeruginosa* bloodstream infections are significant. New strategies are required to treat such infections. We report here the draft genome sequences of two antibiotic-sensitive *P. aeruginosa* bloodstream infection isolates that were associated with rapid death in nonneutropenic patients.

## GENOME ANNOUNCEMENT

In 2011, the Centers for Disease Control and Prevention found that *Pseudomonas aeruginosa* accounted for 7.1% of all health care-associated infections in the United States. In 2014, it was in a tie with another organism as third most common Gram-negative cause of bloodstream infections (BSIs) (1). The mortality rate for *P. aeruginosa* BSI has been found to be up to 42%, depending on the population studied (2). Longitudinal mortality trends related to this infection have shown an increase in mortality over time (3).

Three hundred eighty-eight monomicrobial *P. aeruginosa* BSIs were studied as part of a 3-year multicenter retrospective cohort study. At 48 h after the collection of the sentinel blood culture, 17 patients, representing 17 BSI episodes, had died. The details of this study have been published elsewhere (3). Of these patients, six were neutropenic within the 14-day period prior to the BSI episode and were removed from further study. In one patient, the neutrophil count was not known. Of the remaining 10 BSI episodes, 5 of the BSI isolates had been stored by the servicing laboratory and were available for further study. Antimicrobial susceptibility testing was performed by a microdilution method on the Vitek 2 system. Clinical and Laboratory Standards Institute breakpoints were used to define susceptibility or resistance to aztreonam, ticarcillin-clavulanate, piperacillin-tazobactam, ceftazidime, cefepime, meropenem, ciprofloxacin, and gentamicin (4). All isolates were fully sensitive to the antibiotics tested.

To further study the *P. aeruginosa* BSI isolates associated with rapid patient death, their genomes were sequenced. Paired-end libraries of whole-genome DNA for all five isolates were prepared by the Nextera XT library protocol and sequenced by Illumina HiSeq 2000 or NextSeq 500 (Illumina, San Diego, CA, USA). Sequences were trimmed in CLC Genomics Workbench 7.5.1. All sequences were *de novo* assembled using SPAdes 3.8.1. Annotation was performed using the NCBI Prokaryotic Genome Annotation Pipeline. Two of the isolates had an assembly that had fewer than 300 contigs that were greater than 500 bp in length. The draft genomes were 4,654,913 to 5,287,059 bp long, consisting of 112 to 287 contigs (GenBank accession no. MWZI00000000, 4,563 total genes, 4,512 coding sequences [CDSs], 44 tRNAs, and 4 noncoding RNAs; accession no. MWZG00000000, 5,197 total genes, 5,143 CDSs, 47 tRNAs, and 3 noncoding RNAs). A more detailed report of the virulence genes of these BSI isolates will be included in a future publication.

### Accession number(s).

The draft genome sequences were deposited at GenBank under the accession numbers MWZI00000000 and MWZG00000000.
